# Optimized transcranial direct current stimulation for post-stroke dysphagia with small electrodes: a double-blind, randomized, feasibility study protocol

**DOI:** 10.3389/fneur.2026.1660298

**Published:** 2026-01-29

**Authors:** TaeYeong Kim, Hae-Yeon Park, Sung-Hwa Ko, Yeun Jie Yoo, Hanna Jang, Hyun Mi Oh, Mi-Jeong Yoon, Geun-Young Park, Donghyeon Kim, Tae-Woo Kim, Sun Im

**Affiliations:** 1 Research Institute, NEUROPHET Inc, Seoul, Republic of Korea; 2 Department of Rehabilitation Medicine, Bucheon St. Mary's Hospital, College of Medicine, The Catholic University of Korea, Seoul, Republic of Korea; 3 Department of Rehabilitation Medicine, Pusan National University School of Medicine, Pusan National University Yangsan Hospital, Yangsan, Republic of Korea; 4 Department of Rehabilitation Medicine, St. Vincent's Hospital, College of Medicine, The Catholic University of Korea, Seoul, Republic of Korea; 5 Department of Rehabilitation Medicine, National Traffic Injury Rehabilitation Hospital, Gyeonggi-do, Republic of Korea; 6 Department of Rehabilitation Medicine, Seoul National University College of Medicine, Seoul, Republic of Korea

**Keywords:** MRI-guided therapy, personalized neuromodulation, post-stroke dysphagia (PSD), small electrode, transcranial direct current stimulation

## Abstract

**Rationale:**

Dysphagia affects approximately 78% of post-stroke patients. Transcranial direct current stimulation (tDCS) has demonstrated potential for the treatment of this condition. However, its effectiveness is influenced by individual brain anatomical structure. Also, small-sized electrodes offer significant advantages over conventional larger electrodes by providing increased focality of the electrical field, allowing for precise targeting of cortical regions. Studies that consider both factors are necessary to understand the tDCS effects on this population.

**Aims:**

The present study aims to assess the safety and feasibility of using focalized, optimized tDCS electrode montages in post-stroke dysphagia while considering individual brain anatomy variables improve swallowing function.

**Method and sample size estimates:**

The present study is set to recruit 30 participants, who will be randomly assigned into an active or sham group. Both groups will utilize optimized tDCS electrode positions, determined through computer modeling based on individual magnetic resonance imaging (MRI). Electrode positioning will be calculated to maximize the electric field (E-field) strength within the target region in the swallowing motor cortex, as designated by the investigator on the patient's MRI. The tDCS will be applied for 30 min at 2 mA for 20 sessions using sponge-coated disk electrodes (R = 1.5cm) designed to enhance focality.

**Study outcome(s):**

The primary outcome measurements are the Functional Oral Intake Scale (FOIS) and the Penetration-aspiration Scale (PAS) together with various secondary outcomes, that include the Videofluoroscopic Dysphagia Scale (VDS) and other ancillary parameters that include voice quality and cough strength.

**Discussion:**

We hypothesize that the active tDCS group will demonstrate significant improvements in swallowing function compared to the sham group, establishing the feasibility of personalized, focal-electrode interventions for post-stroke dysphagia rehabilitation.

**Clinical trial registration:**

https://clinicaltrials.gov/ct2/show/NCT06305949, identifier: NCT06305949.

## Introduction and rationale

It has been reported that around 78% of acute stroke patients are prone to develop dysphagia as a consequence of stroke, which is the malfunction of the capacity to swallow (1). Dysphagia severity is related to the stroke location and lesion volume, and additional factors like brain atrophy, white matter changes can further compromise swallowing recovery. Although dysphagia may improve spontaneously with time, approximately 11.5 percent of the patients will develop a long-term disability (2), leading to high mortality and morbidity rate due to several medical complications related to pneumonia, malnutrition, dehydration and recurrent aspiration (1, 3). Therefore, prompt treatment is critical for these patients.

Non-invasive stimulation, particularly transcranial direct current stimulation (tDCS) has drawn attention for post-stroke dysphagia, due to its potential to aid swallowing recovery in addition to its easy application, and low-cost (4) and safety. tDCS uses a constant low-intensity direct current to regulate neuronal activity in the cerebral cortex by positioning electrodes on the scalp (2). Applying a direct current to a specific area of the scalp can modify the threshold of neuronal resting potential and influence the firing of neurons (3). While administering tDCS, the combined effect of the polarity of the electrode (anode or cathode), the intensity and duration of the application can potentially induce depolarization or hyperpolarization of the targeted neurons' membrane (3, 4).

In addition to altering neuronal membrane potentials, accumulating evidence demonstrates that tDCS exerts multi-level effects on the brain. At the cellular level, polarity-specific modulation shifts the excitability of pyramidal neurons, regulates GABAergic interneuron activity, and influences calcium-dependent intracellular signaling cascades, thereby promoting synaptic plasticity (5, 6). At the level of cellular ensembles, tDCS biases spike-timing-dependent plasticity, enhances synchronous discharges, and alters oscillatory activity, which together strengthen functional connectivity across cortical and subcortical swallowing circuits, including the bilateral primary motor cortex, insular cortex, and perisylvian regions (7–9). Converging evidence also indicates that tDCS effects are state- and context-dependent, supporting an interpretation of adaptive network reorganization rather than uniform excitation or inhibition (6). These neurophysiological changes are highly relevant to dysphagia rehabilitation, where restoration of coordinated cortico-bulbar drive is critical, and they align with clinical evidence from systematic reviews and network meta-analyses of non-invasive stimulation for post-stroke dysphagia (10). Cortical oscillatory patterns, particularly in the theta (4–8 Hz), alpha (8–13 Hz), and beta (13–30 Hz) bands, play an important role in swallowing motor control, exhibiting corticomuscular coherence and event-related desynchronization during normal volitional swallowing (11). However, post-stroke dysphagia is associated with disrupted beta band activity and reduced bilateral cortical activation (12). Given that tDCS can modulate cortical oscillatory activity across these frequency bands (13), it may facilitate swallowing recovery through restoration of functional neural synchronization.

Research has shown that when the anode electrode is placed over the pharyngeal motor cortex or the functional brain area in charge of swallowing, the resting membrane potential neurons can be depolarized, increasing the discharge of neurons, and thus enhancing the cortical excitability (4). A study showed that one session of anode tDCS over the pharyngeal motor cortex significantly increased the motor-evoked potential (MEP) amplitude compared to a sham group in a healthy population, increasing corticobulbar motor excitability and resulting in subtle changes in swallowing biomechanics (14).

The potential of tDCS as an added intervention for the treatment of post-stroke dysphagia has been shown in various studies. A study that applied tDCS at 1 mA for 20 min for 4 consecutive days in post-stroke patients found significant improvements in the Functional Oral Intake Scale (FOIS) in contrast to the comparison group (15). Another study applied tDCS at 1 mA for 20 min for 5 days combined with conventional swallowing rehabilitation, finding that the groups receiving tDCS had a significant reduction of the Penetration Aspiration Scale (PAS), showing that tDCS can be used together with conventional therapy for the recovery of patients with post-stroke dysphagia (16).

Although tDCS has shown positive results in post-stroke dysphagia, many studies have employed “one-size-fits-all” approaches without considering individual brain anatomical structure, which has been shown to directly influence the tDCS effects (17). Research has revealed that brain anatomical differences exist even within a particular age group, finding a remarkable inter-individual variability in anatomy at the whole tissue volume/thickness level and cortical morphology (18). These variabilities, combined with the use of large surface electrodes in conventional protocols, influence the electrical field patterns exerted by the tDCS across individuals and therefore its effectiveness (17, 19). Especially in post-stroke patients in which the distribution of induced electric field (E-field) can significantly be affected by the size, shape and location of the lesion (20).

To address these limitations, recent research has explored the use of specialized software (21, 22). This software allows the creation of accurate individualized three-dimensional (3D) brain models for healthy and stroke individuals without the use of extra programs. The software calculates the tDCS induced-E-field according to the individual's brain tissue and stroke lesion characteristics, and tDCS protocol parameters (23). Furthermore, it identifies the electrode position that generates the maximum E-field over a selected target area, which is called the optimized tDCS electrode location.

Computational models based on magnetic resonance imaging (MRI) are valuable for predicting tDCS E-field distribution and determining the optimal placement of electrodes to target the desired brain region while considering individual brain anatomical structure (24). These models create 3D personalized head models that incorporate brain tissue-specified conductivity assumptions and electrode features (size, shape, etc.) (20). Although it has been proven that the 3D head computational models could positively impact the effects of a tDCS intervention, they are not commonly implemented in a clinical application since the creation of these models requires the use of numerous programs and working in collaboration with a computational modeling team (20). Traditional tDCS therapy has limitations due to the variability of individual brain structures, which can lead to inconsistent treatment effects and difficulty in precisely targeting desired brain regions. The software mentioned above helps address these limitations by generating 3D brain models, simulating electrical fields, and calculating optimal electrode placements.

In addition to electrode placement, electrode size influences the efficacy of brain stimulation (25, 26). Small-sized electrodes (1.5 cm radius) offer significant advantages over conventional larger electrodes (5 x 5 or 5 x 7 rectangular electrodes) by providing an increased focality of the electrical field, allowing for precise targeting of cortical regions involved in swallowing function. In contrast, the larger electrodes generate diffuse electrical fields, stimulating broader cortical areas beyond the intended target. This lack of focality may dilute therapeutic effects and increase the likelihood of unintended side effects due to the activation of non-target neural circuits. Furthermore, compared to traditional tDCS protocols, small electrodes reduce the stimulation of non-target areas, potentially improving therapeutic outcomes and reducing adverse events. When combined with the optimized tDCS electrode placement based on individual MRI scans, these small electrodes enable personalized treatment protocols that account for anatomical variability. Small electrodes increase the focality of the electrical field, allow for precise stimulation, reduce side effects, and increase current density, thus giving more effective stimulation.

This double-blind, randomized pilot trial aims to determine the feasibility and safety of optimized transcranial direct current stimulation (tDCS) in patients with post-stroke dysphagia. Specifically, we will (1) evaluate the feasibility of using small-sized and optimized electrode placement to maximize E-field strength in the swallowing motor cortex—comparing active vs. sham stimulation, and (2) assess the clinical safety of this approach in post-stroke dysphagia patients.

## Methods

### Design

**Figure 1:**
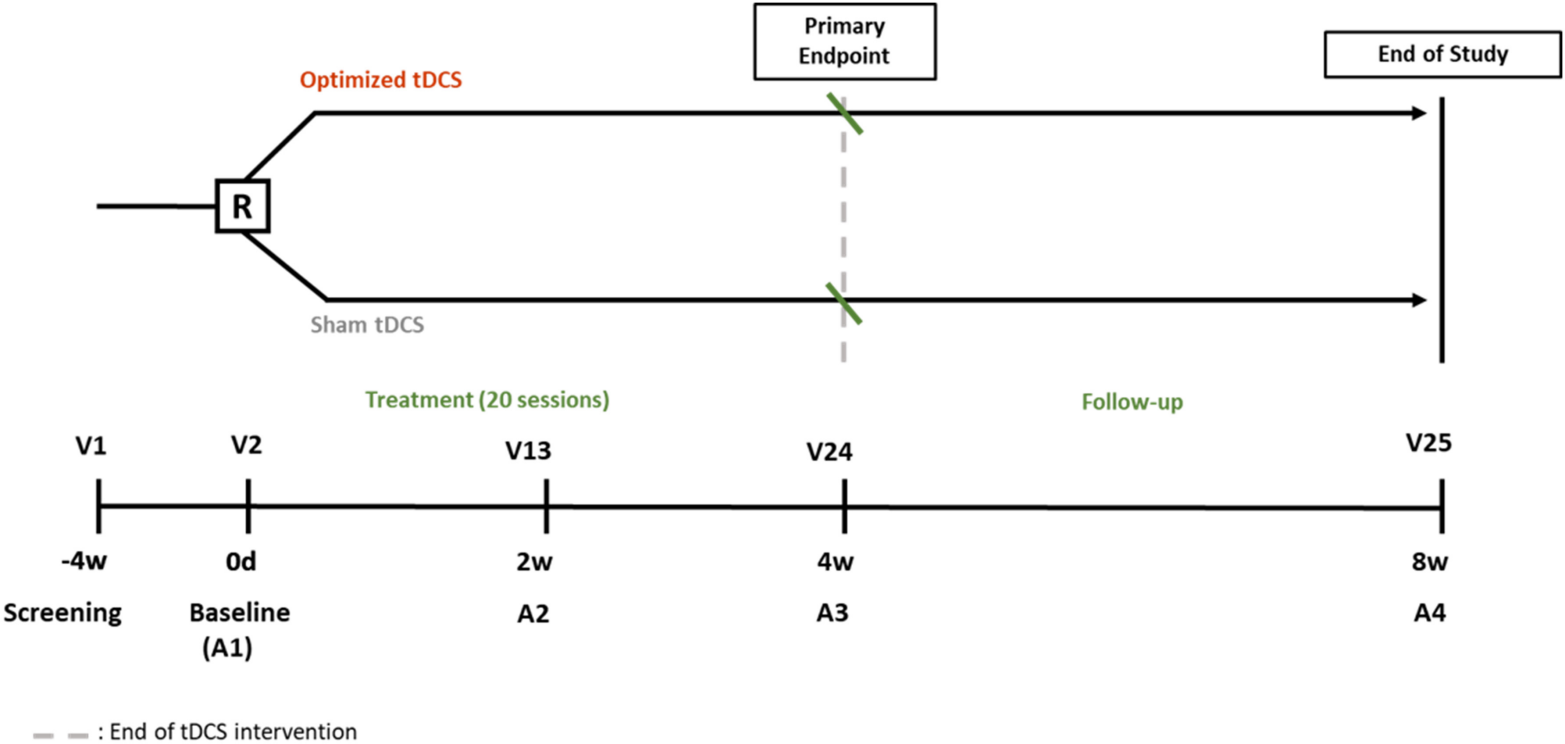
Study design. A, Assessment; R, Randomization; tDCS, Transcranial direct current stimulation; V, Visit.

This multicenter, double-blind, randomized feasibility trial will evaluate the safety and feasibility of optimized tDCS vs. Sham-tDCS in post-stroke dysphagia. Thirty participants will be randomized to receive 20 sessions of active or sham tDCS over 4 weeks. Assessments will be performed at baseline, mid-term, post-intervention and 4-week follow-up (Figure 1).

### Setting and recruitment

Participants will be recruited from four Korean hospitals: Catholic University of Korea Bucheon St. Mary's Hospital, St. Vincent's Hospital, Pusan National University Yangsan Hospital and Seoul National University Hospital, National Transportation Rehabilitation Hospital. Volunteer patients will be screened to determine if they meet the inclusion criteria and none of the exclusion criteria, and then they will be recruited for the intervention, depending on the screening results.

### Patient population and ethics

#### Ethics approval

The study protocol was approved by the Korean Ministry of Food and Drugs Safety (MFDS) and the institutional review boards of all centers (approval numbers: XC23DDDS0098, 24-2023-003, 2023-11-001) and will be conducted in accordance with the Declaration of Helsinki.

#### Inclusion criteria

The volunteers that fulfill the following criteria will be included in the study (1) men and women > 19 years old; (2) patients with stroke confirmed by neuroimaging; (3) first-time stroke patients; (4) patients in subacute or chronic phases of stroke with 3 weeks or more after onset; and (5) stroke patients with confirmed dysphagia through Videofluoroscopic Swallowing Study (VFSS).

#### Exclusion criteria

The volunteers that met at least one of the following criteria will be excluded from the study: (1) patients with recurrent stoke, traumatic brain injury, spine cord injury, and degenerative brain disease, such as Parkinson's disease, etc.; (2) patients with deteriorated cognitive function unable to perform the clinical trial as instructed; (3) patients with evidence of delirium, confusion, or other impairment of consciousness; (4) patients with uncontrolled medical disease or surgical conditions; (5) patients ineligible for tDCS (due to scalp condition, metallic material at the electrode attachment area, presence of a pacemaker or cochlear implant); (6) patients with previous experience within the last year using a stimulation device similar to the one use in this clinical trial or who have participated in related clinical trials; (7) patients with severe neurologic disorder with concomitant major psychiatric disorder such as major depressive disorder and dementia; (8) patients with history of uncontrolled epilepsy within 6 months; (9) patients with medical contraindications for neuroimaging test, such as MRI; (10) patients who are taking contraindicated medications or require dosage adjustments during the trial period that could influence cognitive/motor function changes via brain activation changes; including but not limited to gabapentin, pregabalin, clonazepam, lorazepam, zolpidem tartrate, or other central nervous system (CNS)-acting drugs; (11) patients who are pregnant, breastfeeding, or planning pregnancy during the trial period; and (12) patients considered medically ineligible for participation in the present trial beyond the criteria listened above.

### Randomization

Participants will be randomized using a block randomization method stratified by stroke chronicity (subacute vs. chronic) and age (19–64 vs. ≥65 years). Allocation will be concealed in opaque envelopes; a blinded investigator will assign participants (1:1) without involvement in outcome assessments.

### Intervention

#### Individualized 3D brain modeling and optimized electrode location calculation

Each participant will undergo a comprehensive process to ensure the precise targeting of the swallowing motor cortex using tDCS. To achieve this, individualized 3D brain models will be generated for all participants using the software NEUROPHET tES LAB (Neurophet, Seoul, Republic of Korea). At the baseline of the trial, every participant will take a T1-weighted magnetic resonance imaging (MRI) scan, which will serve as the foundation for the creation of the personalized brain model. The software is designed to analyze and segment the T1-weighted MRI data, thereby constructing a detailed 3D model that accurately reflects the unique anatomical structure of each participant's brain. In addition, the software assigns pre-programmed electrical conductivity values to the various brain tissues, including the skin (0.465 S/m), skull (0.010 S/m), cerebral and cerebellar gray matter (0.276 S/m), cerebral and cerebellar white matter (0.126 S/m), cerebrospinal fluid and ventricle (1.65 S/m) and the stroke lesion area (0.809 S/m), ensuring that the simulation closely mimics the physiological properties of each individual's brain.

Both the Optimized-tDCS and the Sham group will utilize personalized, optimized electrode locations calculated using the NEUROPHET tES LAB. On the 3D brain model of each participant, the investigator will select the target region within the swallowing motor cortex of the non-lesional hemisphere where the E-field is intended to be maximized. Target selection of the swallowing motor cortex target; located in the precentral gyrus, will be made based on convergent evidence from task-based functional MRI studies of volitional swallowing (27). Cortical representation of swallowing exhibits well-documented somatotopic organization: lateral regions correspond to mylohyoid function, while medial zones associate with pharyngeal control (28–30). To balance anatomical specificity with computational feasibility, we will operationalize the target as a 2-mm radius spherical region of interest (ROI) in standardized space, positioned at the centroid between these functional activation sites.

**Figure 2:**
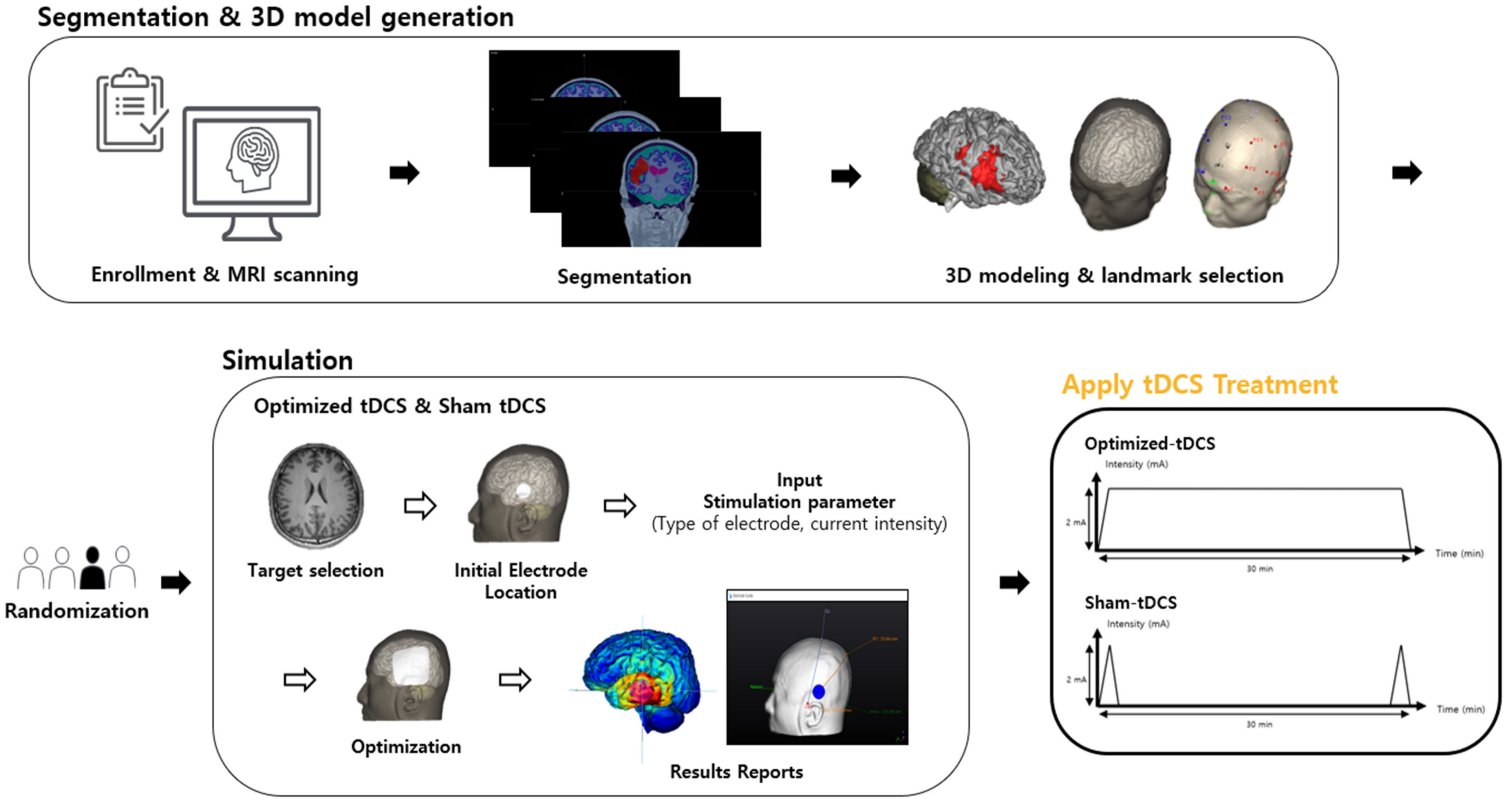
Workflow for Personalized tDCS treatment using NEUROPHET technology.

Within the same software, the investigator will also specify the electrode shape (disk type) and the current intensity (2 mA) to be applied during the stimulation. The software offers a grid-based search function, allowing the investigator to define a search area that covers the region of interest in the swallowing motor cortex. Within this designated area, the software will systematically calculate the optimal positions for the anode and cathode electrodes to maximize the E-field strength at the target site. Once the optimal electrode locations are determined, the software will generate individualized guidance for positioning the electrodes at anatomically optimized scalp locations that correspond to the targeted cortical regions. This process ensures that each participant receives stimulation tailored to their unique brain anatomy, thereby maximizing the potential therapeutic effects while minimizing off-target effects (Figure 2).

#### Transcranial direct current stimulation

For the actual stimulation, the NEUROPHET innk device (Neurophet, Seoul, Republic of Korea) will be employed. This battery-driven, portable tDCS device is programmed to deliver a constant current of 2 mA for 30 min through sponge-coated disk electrodes with a radius of 1.5 cm for the Optimized-tDCS group. To enhance participant comfort and minimize discomfort associated with sudden changes in current intensity, a ramping protocol will be implemented at the beginning and end of each stimulation session. Specifically, the current will be gradually increased to the target intensity over the first 30 s and then gradually decreased at the end of the session over another 30 s. In the case of the sham group, participants will experience the same initial ramp-up and ramp-down of current, but the device will not maintain the current at 2 mA for the duration of the session. This approach ensures that participants in both groups experience similar sensations at the start and end of the stimulation, thereby maintaining the integrity of the double-blind study design.

### Blinding

As a double-blind study, the participants and investigators in charge of assessing the outcomes measurements will remain unaware of the participant's group allocation until the trial has been completed and the data analyzed.

It is challenging to maintain blinding for the medical device investigator, considering the nature of the study of applying personalized tDCS electrode location and the fact that the tDCS device must be programmed according to the assigned group. As expressed before, the medical device investigator will not be involved in other parts of the study.

### Outcome measures

The outcome measurements will be assessed at four points: baseline, Mid-intervention (after finishing the second week of intervention), Post-intervention (after the last tDCS session), and Follow-up (four weeks after the last intervention). Not all the outcomes will be recorded at the mentioned four points; the outcome describes details (Table 1).

**Table 1:** Schedule of enrollment, interventions, and assessments.

**Schedule**	**Screening**	**Baseline**	**tDCS**	**Assessment**	**tDCS**	**Assessment**	
Visit (V)	V1	V2	V3-V12	V13	V14-23	V24	V25
Written consent	V						
Screening number assignment	V						
Demographic information	V						
Medical and surgical history	V						
Inclusion/exclusion criteria screening	V	V					
Vital signs screen	V		V			V	
Physical and medical examination	V	V		V		V	V
Pregnancy test	V						
Laboratory test	V					V	
Assessment of prior concomitant medications	V	V	V	V	V	V	V
Assessment of prior concomitant therapies	V	V	V	V	V	V	V
Randomization		V					
MRI examination	V					V	
tES LAB preparation		V					
VFSS assessment	V	V		V		V	V
FOIS assessment		V		V		V	V
PAS assessment		V		V		V	V
VDS assessment		V		V		V	V
K-MASA assessment		V		V		V	V
GUSS assessment		V		V		V	V
EAT-10 assessment		V		V		V	V
MEP examination		V		V		V	
IOPI assessment		V				V	V
GRBAS scale		V				V	V
Wet voice quality assessment		V				V	V
Respiratory muscle strength measurements		V				V	V
SMST assessment		V				V	V
U-TAP assessment		V				V	V
mRS assessment		V		V		V	V
Pharyngeal stage Assessment		V				V	V
General health and Quality of life assessment		V		V			V
Assessing the cortical E-field		V					
tDCS intervention			V		V		
Adverse events examination			V	V	V	V	V
Satisfaction survey						V	

tDCS, transcranial direct current stimulation; MRI, Magnetic resonance imaging; VFSS, Video fluoroscopic swallowing study; FOIS, Functional Oral intake Scale; PAS, Penetration-aspiration Scale; VDS, Video fluoroscopic Dysphagia Scale; K-MASA, Korean Mann Assessment of Swallowing Ability; GUSS, Gugging Swallowing Screen; EAT-10, Eating Assessment-10; MEP, Motor-evoked potential; IOPI, Iowa Oral Performance Instrument; SMST, Speech Mechanism Screening Test; U-TAP, Urimal Test of Articulation and Phonology; mRS, modified Rankin Scale.

### Primary outcomes

#### Functional Oral intake Scale (FOIS)

The FOIS scale will be assessed as a primary outcome during the baseline and post-intervention periods. FOIS is a reliable and valid ordinal scale widely used for clinical and research purposes to assess changes in functional oral intake of food and liquids in patients with oropharyngeal dysphagia. FOIS is sensitive to changes in oral intake of food and liquid over time in stroke patients. This characteristic is essential when documenting overall stroke recovery or for detailed improvement associated with rehabilitation efforts. The scale ranges from level 1 (nothing by mouth) to level 7 (a full unrestricted oral diet) (31, 32).

#### Penetration-aspiration Scale (PAS)

As a primary outcome, PAS will be assessed at Baseline and Post-intervention. The PAS is a standard scale to assess clinical practice and research deglutition. It is composed of an 8-point scale used to characterize the location of airway invasion events and the patient's response during videofluoroscopic swallowing studies. The scale was designed to capture three constructs: depth of airway invasion, material remaining after swallowing, and patient's response to aspiration. The scale ranges from score 1 (Material does not enter the airway) to score 8 (Material enters the airway, passes below the level of the vocal folds, and no effort is made to eject) (33, 34).

### Secondary outcomes

#### Functional Oral Intake Scale and Penetration-aspiration Scale (FOIS) and PAS

As secondary outcomes, FOIS and PAS will be assessed across 4 outcome periods (Baseline, Mid-intervention, Post-intervention and Follow-up).

#### Videofluoroscopic Dysphagia Scale (VDS)

VDS is known as a reliable predictor of long-term persistent post-stroke dysphagia. The VDS is the objective and quantitative interpretation of the results from a videofluoroscopic swallowing study (VFSS). The scale contains 14 categories that represent both oral functions (lip, closure, mastication, bolus formation, premature bolus loss, apraxia and oral transit time) and pharyngeal functions (pharyngeal triggering, laryngeal elevation, epiglottic closure, pharyngeal transit time, pharyngeal coating, vallecular and pyriform sinus residues, and tracheal aspiration), with a sum of 100 points (35, 36). VDS will be assessed at the past mentioned 4 outcome periods.

#### Korean Mann Assessment of Swallowing Ability (K-MASA)

The K-MASA is the Korean-validated version of the MASA tool, which is a bedside examination for the evaluation of dysphagia that has been validated in first-time stroke patients (37). MASA assesses 24 skills related to sensory and oral motor elements of swallowing. Performance for each skill is measured using a 5- or 10-point rating scale and tallied to create a total numeric score out of 200 possible points, which are interpreted as no abnormality (≥178), mild dysphagia (168–177), moderate dysphagia (139–167) and severe ( ≤ 138) (38). K-MASA will be assessed at the past mentioned 4 outcome periods.

#### Gugging Swallowing Screen (GUSS)

GUSS is a bedside dysphagia screening tool for patients with stroke of easy use for nurses and therapists, that can identify stroke patients with dysphagia and aspiration risk and also can be helpful in choosing dietary prescriptions for the patients. The assessment comprises two parts, the preliminary assessment (indirect swallowing test) and the direct swallowing test, which consist of 4 items with 3 subtests (semisolid, liquid and solid). The test is based on a pointing system where higher numbers denote better performance, with a maximum of 5 points that can be reached in each subtest. Twenty points are the highest score a patient can attain, and it denotes normal swallowing ability without aspiration risk (39). The GUSS will be assessed at Baseline, Mid-intervention, Post-intervention, and Follow-up.

#### Eating Assessment (EAT-10)

The EAT-10 is a tool to assess self-perceived symptoms of oropharyngeal dysphagia and monitor changes in response to a treatment (40). It consists of ten items to be rated on a 5-point response scale (0–4), with “0” = No problem and “4” = Severe problem, the maximum score possible is 40. The validated Korean version of the EAT-10 will be used in this trial (41). The EAT-10 will be assessed at Baseline, Mid-intervention, Post-intervention and Follow-up.

#### Motor-Evoked Potential (MEP)

MEPs are a standard tool for non-invasive quantification of cortical excitability. The cortical excitability of the area corresponding to the swallowing movement will be assessed by changes in the amplitude and latency of the MEPs by using transcranial magnetic stimulation (TMS). The MEP responses of the mylohyoid and thenar musculature will be recorded. The MEP will be induced by the application of TMS over the motor cortex, the coil will be placed at an angle of 45° to the parasagittal plane, tangential to the scalp surface, since this is the ideal orientation for evoking both swallow and thenar muscle response, the stimuli will be applied bilaterally (42). Resting motor thresholds, amplitude, and latency of evoked potentials will be measured before and after the TMS brain stimulation. MEP will be assessed during Baseline, Mid-intervention and post-intervention.

### DTI-based assessment of corticobulbar tract

The corticobulbar tract's white matter integrity is crucial for optimal swallowing function, with empirical evidence showing that damage to these tracts significantly exacerbates dysphagia severity (43, 44). Moreover, white matter demonstrates a high capacity for neuroplastic potential, as structural and functional reorganization can be observed following targeted neuromodulator interventions. Therefore, we will conduct a comprehensive quantitative analysis to systematically evaluate tDCS-induced neuroplastic changes. Specifically, diffusion tensor imaging (DTI) will be acquired at baseline and post-intervention, enabling 3D tract reconstruction and quantitative assessments (e.g., fractional anisotropy, mean diffusivity) of the corticobulbar tract.

### Iowa Oral Performance Instrument (IOPI)

The IOPI is a standardized portable device that is used to quantify tongue and lips isometric strength and endurance. It consists of a pressure transducer and an amplifier that displays on a screen, in kilopascals (kPa), the pressure exerted on an air-filled bulb. The device can measure the pressures applied by the tongue on the bulb after inserting the air-filled balloon inside the mouth and pressing the bulb against the roof of the mouth. The lips exerted pressure in kPa, can be obtained by pressing the air-filled bulb between the lips (45, 46). The IOPI will be assessed only at the Baseline, Post-intervention, and Follow-up.

### Ancillary respiratory stage assessment

The ancillary respiratory stage assessment will be performed to assess the function of the pharyngeal phase. The voice quality will be assessed by the GRBAS (grade, roughness, breathiness, asthenia, strain) scale (47) and Wet voice quality (48). The respiratory muscle strength will be assessed by measuring the maximal inspiratory and expiratory pressure using spirometry (49, 50); and the cough strength will be evaluated by the voluntary peak cough flow (51). These parameters will be assessed only at the Baseline, Post-intervention and Follow-up.

### Speech Mechanism Screening Test (SMST)

The SMST evaluates the oral function by assessing the structure and function of the articulatory system, the coordination process, and the maximum speed, regularity, and accuracy of the muscles that make up the articulatory system. The test consists of 13 questions to evaluate the structure of the articulatory system, including the face, lips, tongue, jaw, teeth, oral cavity, soft palate, pharynx, and breathing; 17 questions to evaluate the function of the articulatory system; 3 questions to auditorily evaluate the structure and function of the vocal system; and 14 questions to evaluate the regularity and articulation accuracy of the mutual movement of the articulatory system during articulatory alternation. The phonation-voice and articulation screening assess the maximum phonation time (MPT) for the/a/vowel. It assesses the pitch, loudness, speech rate, and voice quality during spontaneous name and address production and reading of a given sentence. Articulation errors and alternating movement rate (AMR) and serial movement rate (SMR) are measured to screen for functional or neurological coordination system abnormalities in articulation production, and test items in each area are scored and evaluated (52). The SMST will be assessed only at Baseline, Post-intervention and Follow-up.

### Urimal Test of Articulation and Phonology (U-TAP)

U-TAP is a test designed for native speakers of the Korean language. It is aimed at evaluating the pronunciation produced in words and sentences by patients with speech sound disorders who have problems with consonants or vowel sounds. It is recorded by phonological words and syllable position and calculates consonant accuracy and word-level phonological indices (PWC, PMLU, PWP) at the word and sentence levels to compare with normal articulation development (53). The U-TAP will be assessed only at Baseline, Post-intervention, and Follow-up.

### modified Rankin Scale (mRS)

mRS is used to assess the changes in activity lifestyle after stroke. It is composed of a 6-point assessment that includes reference to both activity limitations and lifestyle changes. The mRS possible scores vary from 0 (no symptoms) to 6 (dead) points (54). The mRS will be assessed at the Baseline, Mid-intervention, Post-intervention, and Follow-up.

### Quality of life index

The EuroQoL five-dimensional instrument with five levels (EQ-5D-5L) will assess the participants' quality of life. EQ-5D-5L consists of five dimensions of health: mobility, self-care, usual activities, pain/discomfort, and anxiety/depression. Each has five levels or response categories: no problem, slight problems, moderate problems, severe problems and extreme problems (55). The health states are defined by combining one level from each of the five dimensions, for 3,125 possible health states. For example, “11111” indicates no problems on the five dimensions. In contrast, “11125” indicates no problems with mobility, self-care, and usual activities, but slight issues with pain/discomfort and extreme anxiety/depression. The questionnaire has been validated for the general Korean population (55). The EQ-5D-5L will be assessed at Baseline, Mid-intervention, Post-intervention and Follow-up.

### General health

A general health examination will be performed during the four mentioned periods. The examination will be composed of a combination of various tests, which include nutritional examination (blood sample recollection of albumin and protein) (56), evaluation of the presence of low skeletal muscle index (56, 57), and immunologic tests (number and type of infections, white blood cell count and antibiotic use history) (58).

### Correlation between cortical E-field over the stimulated area and MEP, FOIS and PAS

To determine if there is a lineal association between the E-field generated at the Baseline by the Optimized electrode location and the results of the MEP, FOIS and PAS.

### Sample size estimates

Due to the feasibility nature of the study, the primary goal is to evaluate the safety, practicability, and preliminary effects of optimized tDCS stimulations with personalized electrode placement in patients with post-stroke dysphagia. Consistent with established guidelines for feasibility and pilot studies, a formal power calculation for efficacy was not performed. Instead, the sample size of 30 participants (15 per group) was determined based on practical considerations and precedent from previous studies in this field. Specifically, a similar previous study (16) demonstrated protocol feasibility and observed clinically relevant improvements in swallowing function among 28 participants (14 per group) undergoing a 20-session tDCS intervention. Our study adopts a comparable intervention schedule and aims to provide reliable estimates of key feasibility outcomes-including recruitment rates, adherence, and adverse event profiles-while also generating preliminary data on the variability and potential effect size of our primary and secondary outcome measures. These findings will be essential for informing the design and sample size calculation of a future, large-scale randomized controlled trial.

### Statistical analysis

The study outcomes will be presented as mean, standard deviation (SD), median, minimum, and maximum for continuous data and as frequencies and proportions for categorical data. The changes within groups from the baseline to the outcome measurement periods, according to each outcome, will be assessed with paired *t*-test (or Wilcoxon's rank sum test if the data do not meet the normality assumption). Between-group changes at each time point will be assessed by a two-sample *t*-test (or a Wilcoxon rank sum test will be used if normality is not met). Repeated measures analysis of covariance (ANCOVA) will assess group differences across time points, using baseline as a covariate. In addition, subgroup analyses will be conducted by age (19–64 and 65+).

For categorical variables, Pearson's chi-square test (or Fisher's exact test if the number of cells with an expected frequency of < 5 exceeds 20%) will be applied, and McNemar's test will analyze intra-group comparisons. In the case of physical examination, the results at each time point will be categorized as Normal, Abnormal Not Clinically Significant, and Abnormal Clinically Significant and the frequencies will be reported. For the concomitant medications, the number of participants who were taking concomitant medications, the percentage, and the number of doses will be reported.

### Safety and adverse event monitoring

Adverse events will be systematically assessed and recorded by investigators throughout the trial, including during and up to 30 min after each tDCS session. Predicted adverse events specific to tDCS include itching, tingling, headache, burning, and discomfort at the stimulation site. In addition, adverse events related to post-stroke dysphagia-such as the pneumonia, enteritis, fever and abnormal breath sounds-will also be monitored.

All adverse events will be categorized as adverse device events (ADEs), serious adverse events (SAEs), and serious adverse device events (SADEs) and their incidence, number, and percentage per event will be documented. Events will be further standardized using the most recent version of the Medical Dictionary for Regulatory Activities (MedDRA), with reporting by System Organ Class (SOC) and Preferred Term (PT). These data will be analyzed and reported by group to ensure comprehensive safety monitoring throughout the study.

### Vital signs and physical examination

The vital signs will be assessed after 5 min of resting in a seated position by measuring systolic and diastolic blood pressure, pulse and body temperature. They will be assessed at Baseline, Mid-intervention and post-intervention.

The physical examination will be performed at Baseline, Mid-intervention, Post-intervention, and Follow-up. It includes assessing height, weight, skeletal muscle index, body mass index, body water, protein, minerals, and body fat percentage.

### Concomitant medications and clinical treatments

There are no specific restrictions on medication usage for the current trial. However, to minimize the impact of any medication on the intervention, the participants who are consuming medication must remain with a stable and unaltered treatment prescription for at least 1 week before screening to allow concomitant medication administration. Additionally, changes in the type of medication and dosage during trial participation are not allowed.

However, as some medications might affect the cough reflex (sedatives, pain medications, etc.), caution will be implemented before performing the VFSS test. A detailed record of the type of medication, dosage, and reason for taking the medication will be documented.

Swallowing rehabilitation treatment can be maintained during the clinical trial without interruption. The participant might be allowed to receive concomitant surgery, medication, or other medical device interventions that are not considered to influence the interpretation of the results. The method and number of swallowing rehabilitation treatments received during the clinical trial, the types and dosages of all concomitant medicines, etc. will be documented at each visit by the investigator and recorded in the case report form (CRF), as well as confirmed adverse events.

### Study organization and funding

This research was supported by a grant of the Korea Health Technology R&D Project through the Korea Health Industry Development Institute (KHIDI), funded by the Ministry of Health & Welfare, Republic of Korea (grant number: HI23C0584).

### Trial status

The recruitment of the participants is set to begin in March of 2024, and it is expected to be complete (including Follow-up testing) by May 2026.

## Discussion

The purpose of this multi-center trial is to determine the feasibility and safety of optimized tDCS stimulations with optimized electrode placements to improve swallowing function in patients with post-stroke dysphagia. By utilizing individualized three-dimensional brain models based on MRI scans, we aim to optimize electrode placement. Employing small-sized electrodes (1.5 cm radius) we further aim to increase the focality of the electrical field, allowing for precise targeting of cortical regions involved in swallowing function. This comprehensive, multi-center trial, involving four participating centers and stratifying patients based on age, chronicity, and center, aims to increase the generalizability of our findings. The assessment of a variety of dysphagia-related outcomes will provide a comprehensive evaluation of the treatment's impact. The integration of computational modeling with the application of small-sized electrodes presents an innovative strategy that could significantly improve rehabilitation outcomes for patients with post-stroke dysphagia.

Dysphagia significantly impacts the quality of life and increases mortality rates in post-stroke patients (4, 16). According to research, tDCS has the potential to become an added intervention for the improvement of swallowing dysfunction (4, 59). However, systematic reviews indicated that multicenter randomized controlled trials (RCT) with large populations are needed to draw more accurate conclusions about the effect of tDCS on the improvement of dysphagia, but especially to determine the best tDCS protocol to achieve it (4). As a multicenter RCT focused on optimizing tDCS electrode positions, the present study is crucial for the future use of tDCS in dysphagia rehabilitation.

Past research has used anodal tDCS over the lesioned side to treat dysphagia, finding improvement in the swallowing function (60, 61). Targeting the ipsilesional side might increase the activity of the lesional hemisphere and decrease the transcallosal inhibition exerted by the contralesional hemisphere, which is believed to be hyperactive after stroke (61). Additionally, some studies have suggested that targeting the ipsilesional motor region could reactivate intact portions of the motor cortex (62). Other research has shown that after stroke, compensatory reorganization of the contralesional hemispheric projections influences the recovery of swallowing function and therefore, some studies have applied anodal stimulation to the contralesional side (15, 63). Both anode locations have been shown to affect the improvement of the swallowing function positively. Based on evidence from Suntrup et al. (64), that post-stroke dysphagia recovery involves neuroplasticity in the intact hemisphere, we will apply anodal tDCS to the non-lesional hemisphere to enhance swallowing function.

While current research has shown the potential of tDCS for improving swallowing dysfunction in post-stroke dysphagia patients, to the best of our knowledge, research has yet to consider individual brain anatomical structure when determining the optimal tDCS electrode placement for tDCS intervention protocols in these patients. According to research, the conventional approach involves using the 10–20 EEG system to position tDCS electrodes over the cortical swallowing area, typically by identifying the midpoint between C3 and T3 or C4 and T4 (depending on the lesioned hemisphere) to stimulate the swallowing cortex. However, the 10–20 EEG system placements, although approximations to the targeted area, do not account for inter-individual variations in brain structures, such as skull thickness and gray and white matter integrity (65). Calculating an optimized electrode location offers a personalized electrode setting per participant, giving the exact location predicted to generate the maximum E-field over a targeted area.

In addition to the electrode location, tDCS effects are believed to be influenced by electrode shape and size. The majority of tDCS studies in patients with post-stroke dysphagia typically employed 25 cm^2^ electrode size (16, 60, 66, 67). Computer simulation studies evidenced that larger electrodes tend to stimulate broader brain regions, resulting in a dispersed current density. Furthermore, these electrodes contribute to the edge effect, leading to a concentration of the current at the edges, notably at the corners of rectangular and square electrodes (68). Employing smaller, circular-shaped electrodes may mitigate the edge effects and refine the precision of the current targeting at the intended area; therefore, disk electrodes of 1.5 cm will be used for the present trial.

For the present study protocol, we hypothesize that the optimized-tDCS group will show significant improvement in swallowing function compared to the Sham group, demonstrating the feasibility of using optimized electrode locations for tDCS stimulation for post-stroke dysphagia. Additionally, we hypothesize that all the participants will complete the intervention without any significant adverse effects after using optimized tDCS electrode location.

This study is significant in its assessment of the safety and efficacy of a personalized, focal tDCS approach using small-sized electrodes guided by each patient's individual brain computational modeling. By optimizing electrode placement for each participant, we aim to enhance both the therapeutic precision and safety of tDCS for post-stroke dysphagia. There are some potential limitations to disclose. First, there is currently no prior clinical evidence directly validating the safety of the specific electrode size proposed in this study. However, a preliminary computational model comparing E-field strengths using different size electrodes is being carried out (69). Also, the findings of this clinical trial will help address this gap by providing preliminary safety data. Second, while this is an exploratory trial, its value lies not only in demonstrating feasibility and safety, but also in establishing a foundation for a subsequent pivotal trial aimed at evaluating therapeutic efficacy on a larger scale. Additionally, our study included both ischemic and hemorrhagic stroke patients without subtype stratification. While our study included both ischemic and hemorrhagic stroke patients without subtype stratification, we deem that this approach was appropriate for a pilot feasibility study and reflects real-world clinical heterogeneity, though future definitive trials may benefit from pre-planned subtype stratification based on these preliminary findings. Our stratification by stroke onset time and age addresses more mechanistically relevant predictors of tDCS response than stroke etiology, particularly given our non-lesional hemisphere targeting approach. Furthermore, this mixed population methodology is consistent with major systematic reviews and meta-analyses that routinely treat stroke as a homogeneous condition without subgroup analysis by etiology (3, 4) and is supported by evidence showing comparable tDCS safety profiles between stroke subtypes. Ultimately, the outcomes of this study will inform future clinical trials and contribute to the development of more effective, personalized neurostimulation treatments.

## Summary and conclusions

Post-stroke dysphagia is a common complication which may adversely affect patients' long-term prognosis. Although tDCS has demonstrated therapeutic potential in prior studies, its efficacy may be further enhanced through personalized and optimized stimulation techniques. This study protocol introduces a novel approach with individualized electrode placement, refined cortical targeting, and a rigorous methodological framework. By addressing limitations in earlier trials and integrating computational modeling and focal stimulation techniques, the study aims to improve swallowing outcomes in individuals with post-stroke dysphagia and establish tDCS as a clinically viable treatment strategy. Findings from this study will provide critical data to support the planning and implementation of larger, multi-center RCT.
